# Co-Circulation of Multiple Serotypes of Bluetongue Virus in Zambia

**DOI:** 10.3390/v12090963

**Published:** 2020-08-31

**Authors:** Herman M. Chambaro, Michihito Sasaki, Edgar Simulundu, Isaac Silwamba, Yona Sinkala, Gabriel Gonzalez, David Squarre, Paul Fandamu, Caesar H. Lubaba, Musso Munyeme, Alikhadio Maseko, Choopa Chimvwele, Liywalii Mataa, Lynnfield E. Mooya, Andrew N. Mukubesa, Hayato Harima, Kenny L. Samui, Hetron M. Munang’andu, Martin Simuunza, King S. Nalubamba, Yongjin Qiu, Michael J. Carr, William W. Hall, Yuki Eshita, Hirofumi Sawa, Yasuko Orba

**Affiliations:** 1 Research Center for Zoonosis Control, Hokkaido University, Sapporo 001-0020, Japan; m-sasaki@czc.hokudai.ac.jp (M.S.); harima@czc.hokudai.ac.jp (H.H.); h-sawa@czc.hokudai.ac.jp (H.S.); 2 Virology Unit, Central Veterinary Research Institute, Lusaka 10101, Zambia; emmamooya@yahoo.com; 3 Ministry of Fisheries and Livestock, Lusaka 10101, Zambia; sinkalay@yahoo.co.uk (Y.S.); pfandamu@gmail.com (P.F.); caesar.lubaba@gmail.com (C.H.L.); alikhadio21@yahoo.co.uk (A.M.); chimvwele@yahoo.co.uk (C.C.); lmataa_nmataa@yahoo.com (L.M.); 4 School of Veterinary Medicine, The University of Zambia, Lusaka 10101, Zambia; isaacsilwambak@gmail.com (I.S.); mussomunyeme@gmail.com (M.M.); mukubesaandrew@gmail.com (A.N.M.); kennysamui@yahoo.com (K.L.S.); martin.simuunza@unza.zm (M.S.); king.nalubamba@unza.zm (K.S.N.); 5 Macha Research Trust, Choma 10101, Zambia; 6 National Virus Reference Laboratory, School of Medicine, Dublin DO4V1W8, Ireland; gabo.hokudai@gmail.com (G.G.); michael.carr@ucd.ie (M.J.C.); william.hall@ucd.ie (W.W.H.); 7 The University of Edinburgh, Edinburgh EH25 9RG, Scotland, UK; davidsquarre@yahoo.co.uk; 8 Department of National Parks and Wildlife, Chilanga 10101, Zambia; 9 Faculty of Veterinary Medicine and Biosciences, Norwegian University of Life Sciences, 0454 Oslo, Norway; hetroney.mweemba.munangandu@nmbu.no; 10 Hokudai Center for Zoonosis Control in Zambia, School of Veterinary Medicine, University of Zambia, Lusaka 10101, Zambia; yongjin_qiu@czc.hokudai.ac.jp (Y.Q.); yeshita@czc.hokudai.ac.jp (Y.E.); 11 International Collaboration Unit, Research Center for Zoonosis Control, Hokkaido University, Sapporo 001-0020, Japan; 12 Global Virus Network, Baltimore, MD 21201, USA

**Keywords:** *Reoviridae*, bluetongue, bluetongue virus, serotypes, topotypes, sero-surveillance, wild ruminants, domestic ruminants, Zambia

## Abstract

Bluetongue (BT) is an arthropod-borne viral disease of ruminants with serious trade and socio-economic implications. Although the disease has been reported in a number of countries in sub-Saharan Africa, there is currently no information on circulating serotypes and disease distribution in Zambia. Following surveillance for BT in domestic and wild ruminants in Zambia, BT virus (BTV) nucleic acid and antibodies were detected in eight of the 10 provinces of the country. About 40% (87/215) of pooled blood samples from cattle and goats were positive for BTV nucleic acid, while one hartebeest pool (1/43) was positive among wildlife samples. Sequence analysis of segment 2 revealed presence of serotypes 3, 5, 7, 12 and 15, with five nucleotypes (B, E, F, G and J) being identified. Segment 10 phylogeny showed Zambian BTV sequences clustering with Western topotype strains from South Africa, intimating likely transboundary spread of BTV in Southern Africa. Interestingly, two Zambian viruses and one isolate from Israel formed a novel clade, which we designated as Western topotype 4. The high seroprevalence (96.2%) in cattle from Lusaka and Central provinces and co-circulation of multiple serotypes showed that BT is widespread, underscoring the need for prevention and control strategies.

## 1. Introduction

Bluetongue (BT) is a non-contagious, but infectious notifiable arthropod-borne viral disease affecting wild and domestic ruminants, camelids and occasionally large carnivores [1,2]. It is caused by the BT virus (BTV), a member of the family *Reoviridae*, genus *Orbivirus*. Although sheep and white-tailed deer are highly susceptible to BTV infection, cattle and goats remain largely asymptomatic [3]. Some BTV strains, such as the virulent serotype 8, are known to cause clinical disease in cattle [4,5]. Asymptomatic cattle with prolonged viremia are considered to play an important role in the epidemiology of BT [6].

BTV possesses a double-stranded RNA genome consisting of 10 segments that encode 7 structural (VP1–VP7) and 5 non-structural proteins (NS1, NS2, NS3/NS3a, NS4 and NS5) which are packaged in a three-layered icosahedral protein capsid [7,8,9]. The outer capsid layer is composed of VP2 and VP5 proteins, encoded by genome segments 2 and 6, respectively. The VP2 and VP5 proteins induce neutralizing antibodies with the former being the major determinant of virus serotype [7]. To date, 28 BTV serotypes have been identified based on serum neutralization assays and/or nucleotide sequence analysis of segment 2 gene [10,11,12]. Additionally, segment 2 and 6 of different BTV strains can be classified into distinct nucleotypes, of which, 12 segment 2 (A to L) and 10 segment 6 (A to J) nucleotypes have been reported thus far [13,14,15]. Sequence analysis of other genome segments, such as segment 10 and 7, allows the assignment of BTV strains into either Western or Eastern topotypes, which can be further divided into minor subgroups that reflect their geographical origins [15]. Due to the high genetic diversity of segment 2, there is phylogenetic incongruency between segment 2 and 10.

BTV is transmitted primarily by a few species of adult female *Culicoides* midges. In Africa, *Culicoides imicola* and *Culicoides bolitinos* are the principal vectors [16,17]. Even though BT is generally considered endemic to Africa, there is a paucity of information on its occurrence in most countries. Usually, BT outbreaks coincide with periods of high rainfall [18]. In North and East Africa, BT outbreaks involving cattle, sheep and goats have been reported in Egypt, Algeria, Tunisia and Kenya (https://www.oie.int/wahis). In Southern Africa, outbreaks in sheep and goats have been reported in Botswana, Lesotho, Madagascar, Namibia, South Africa and Zimbabwe (https://www.oie.int/wahis). However, in Southern Africa, information on circulating serotypes is only available for South Africa (serotypes 1 to 24) and Malawi (serotypes 1, 2, 3, 5, 8, 10, 15, 20, 21 and 22) [18,19]. In Zambia, apart from limited serological evidence of BT in domestic and wild ruminants conducted over three decades ago, there is no information on the distribution of the disease and circulating serotypes [20,21,22]. Similarly, there is very little information on the ecology and distribution of *Culicoides* midges in Zambia [23].

Zambia is divided into 10 administrative provinces and 117 districts. The country is landlocked and shares its borders with eight countries in Southern Africa. According to data obtained from the Ministry of Fisheries and Livestock, regulated trade in livestock and livestock products between Zambia and other countries in Southern Africa is high. However, the porous nature of Zambia’s borders facilitates unregulated trade and migration of wildlife through regional corridors. Similarly, the complex and largely unregulated nature of most borders in Southern Africa implies that local disease outbreaks can have regional socio-economic consequences [24]. Transboundary animal diseases such as foot and mouth disease and African swine fever have been shown to spread across regional borders during disease outbreaks [24,25]. Equally, the absence of regional BT prevention and control strategies possess a risk for disease spread by regulated and unregulated trade and natural migration of wildlife through regional corridors.

As of January 2018, the total cattle, goat and sheep population in Zambia was estimated to be 7.5 million, of which, the small-scale or traditional sector accounted for 96% of the total population (https://www.zamstats.gov.zm). The majority of small-scale farmers in Zambia rear locally adapted indigenous cattle, goats and sheep. In spite of this, data from the Ministry of Fisheries and Livestock indicate that in the last decade, there has been a significant increase in importation of breeding stock such as cattle (dairy), goats and sheep. This has been a result of government efforts to diversify the economy to support agriculture. While diseases such as foot and mouth disease have drastically affected poor small-scale farmers who largely depend on livestock as a source of protein, income and draft power [26], the socio-economic effects of BT in Zambia are not understood, largely due to the lack of disease surveillance mechanisms. In this study, molecular and serological evidence of the presence of BT in Zambia is reported. The findings have implications for evidenced-based formulation of prevention and control strategies.

## 2. Materials and Methods

### 2.1. Study Area

**Figure 1:**
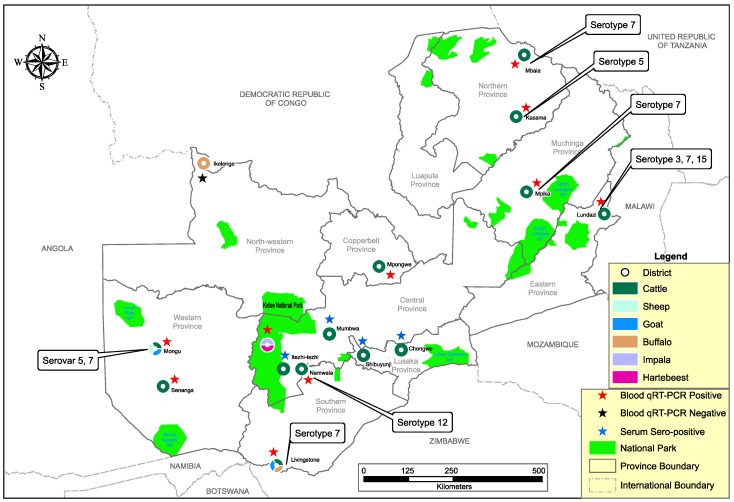
Map showing sample collection sites, bluetongue virus nucleic acid detection, antibody and serotyping results in selected districts and provinces of Zambia.

The country-wide investigation into the epidemiology of BT was triggered by the unexpected detection of BTV nucleic acid in a blood-engorged *Culex quinquefasciatus* mosquito pool captured during routine surveillance for arboviruses in Zambia. We collected whole blood (*n* = 679) from domestic and wild ruminants between August 2018 and December 2019. Of these, 603 samples were collected from traditional cattle, sheep and goats in Southern, Western and Copperbelt provinces, while 76 samples were obtained from wildebeest at a private game ranch in Lusaka Province. Archived whole blood (*n* = 1674) collected from indigenous cattle and wild ruminants (i.e., buffalo, impala and hartebeest) between January 2016 and May 2018 were included in this study. Stored sera (*n* = 449) collected from traditional cattle in Lusaka and Central Province in March 2017 were used to screen for antibodies against BTV. In summary, a total of 2802 samples from 14 districts in nine of the 10 provinces of Zambia were analyzed (Figure 1 and Table 1). Ethical considerations; This study was commissioned and approved by the Ministry of Fisheries and Livestock of the Government of the Republic of Zambia (MFL/DVS/05-02-18).

### 2.2. Blood and Serum Samples

Blood (*n* = 2353; Table 1) was pooled (*n* = 259) as described previously [27] and total RNA was extracted using the QIAamp Viral RNA Mini Kit (https://www.qiagen.com) according to the manufacturer’s protocol. Initial screening for BTV nucleic acid was conducted using a real-time reverse transcription (qRT-PCR) assay targeting segment 9 of BTV genome [28]. Samples with cycle threshold values below 39 were classified as positive based on parallel results obtained by the World Organisation for Animal Health (OIE) recommended nested RT-PCR assay (https://www.oie.int). In pools classed as positive by qRT-PCR, RNA was extracted from individual samples that constituted the pool and screening for BTV genome was conducted by qRT-PCR assay [28]. For serotype determination, a partial region of the segment 2 genome was amplified using PrimeScript One Step RT-PCR kit (Takara, Shiga, Japan) as previously described [29]. To amplify segment 10 gene which is used in topotype determination [14], primers (sense primer; 5′-ATGCTATCCGGGCTGATYC-3′ and antisense primer; 5′-CCCGYTAKACACARCAGTRGG-3′) were designed in Geneious software (https://www.geneious.com) and RT-PCR was performed using PrimeScrip One Step RT-PCR kit as previously described [30].

### 2.3. Sequencing and Phylogenetic Analysis

Sequence libraries for segments 2 and 10 were prepared using Illumina Nextera XT DNA Library Preparation Kit and sequenced on illumina Miseq (https://www.illumina.com). Raw reads were assembled *de novo* in Geneious software and phylogenetic analyses were implemented in MEGA 7 (https://www.megasoftware.net). Nucleotide sequences were deposited in the DDBJ GenBank under accession numbers LC569997-LC570005 and LC570261-LC570271.

### 2.4. Serologic Analysis

Cattle sera were analyzed for BTV antibodies using the ID Screen Bluetongue Competition ELISA assay (IDVet, Grabels, France), which is based on the VP7 protein of BTV as per manufacturer’s instructions. Samples with a competition percentage ≥ 40% were considered negative while those <40% were considered positive. Samples that gave inconclusive results were re-tested. Prevalence of BTV nucleic acid and antibodies were calculated using EpiTools epidemiological calculators (https://epitools.ausvet.com.au).

## 3. Results

### 3.1. Molecular Screening and Phylogenetic Analysis

Of the 259 pooled blood samples analyzed by qRT-PCR, 87 (33.6%) were positive for BTV nucleic acid (Table 2). Overall, BTV nucleic acid was detected in domestic ruminants from all study sites (districts) in Eastern, Western, Southern, Northern, Muchinga and Copperbelt provinces, while only a single hartebeest pool from Kafue National Park in Central Province was positive among the wildlife samples (Figure 1 and Table 2). The overall estimated pooled prevalence of BTV nucleic acid was 4.4% (95% CI (3.6–5.4)) (Table 3). Pooled prevalence in cattle was 4.8% (95% CI (3.8–6.0)), slightly lower than what was observed in goats (7.2%, 95% CI (3.3–13.1)). In wildlife, pooled prevalence was 0.4% (95% CI (0.0–1.8)), significantly lower (*p* < 0.001, OR = 0.04) than in cattle (4.8%) and goats (7.2%). Prevalence in the dry season (2.2%; 95% CI (1.4–3.2)) was significantly low (*p* < 0.0001, OR = 5.4) as compared to the wet season (7.3%; 95% CI (5.6–9.3)). In cattle and goats, the pooled prevalence for the dry season was 1.8% (95% CI (1.0–3.1)) and 2.7% (95% CI (1.7–4.0)), respectively.

**Figure 2:**
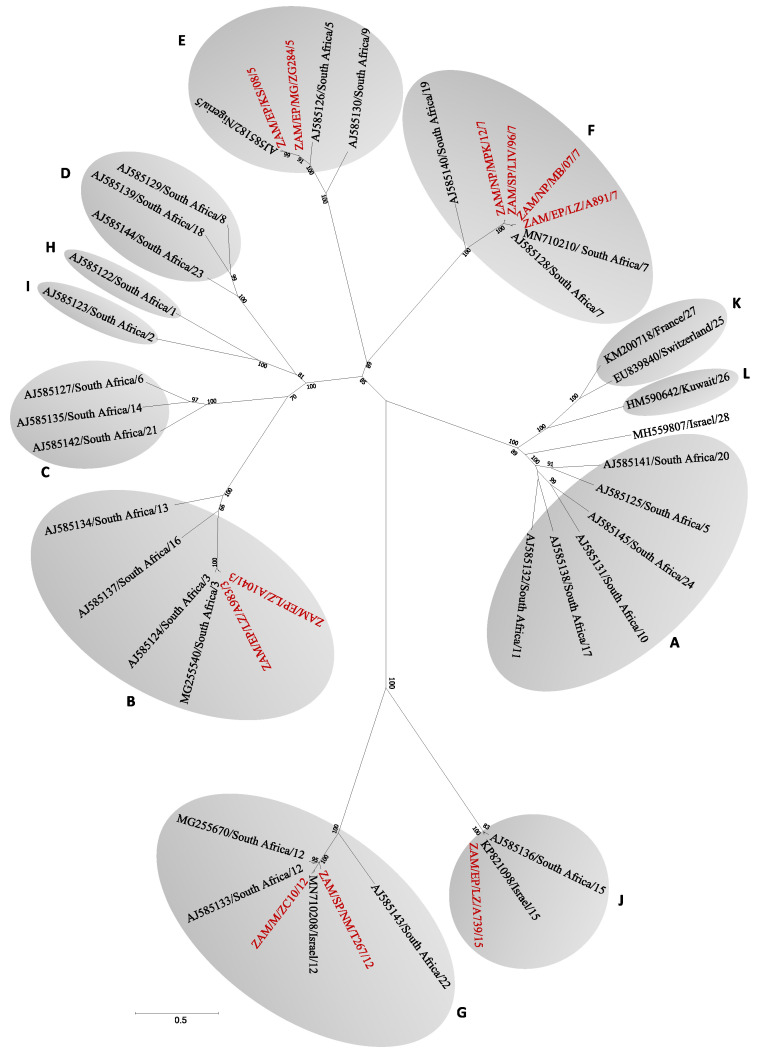
Phylogenetic tree of segment 2 gene of viruses detected in this study. The tree was generated using the Maximum Likelihood method based on the general time reversable (GTR) model with 1000 bootstrap replicates. Numbers at branch nodes indicate bootstrap values (>60%). Viruses characterized in the present study are in red text. Shaded area and letters represent BTV nucleotypes. References sequences for BTV1 to 28 are in black text. Bar—number of nucleotide substitutions per site. Abbreviations: EP, Eastern Province; SP, Southern Province; WP, Western Province; NP, Northern Province; LZ, Lundazi; MG, Mongu; LIV, Livingstone; NM, Namwala; MB, Mbala; KS, Kasama; MPK, Mpika.

On sequence analysis of segment 2, five different serotypes were identified (i.e., 3, 5, 7, 12 and 15) (Figure 1). Serotypes 5 and 7 were detected in Northern Province, while in Eastern Province, serotypes 3, 7 and 15 were identified. In Western Province, we detected serotypes 5 and 12, whereas in Southern Province, serotypes 7 and 12 were identified. Repeated attempts to identify circulating serotypes in other areas were unsuccessful. On BLAST search, serotype 7 viruses shared the highest nucleotide identity (93.4–95.1%) with an isolate from South Africa (accession no. MN710210), while serotype 5 viruses from Kasama (KS08) and Mongu (ZG284) were similar to Nigerian (96.4%; accession no. AJ585182) and Australian (92%; accession no. MG924987) isolates, respectively. Serotype 3 viruses shared close similarity (98.2–99.4%) to an isolate from South Africa (accession no. MG255540), while serotype 12 viruses from Namwala and Mongu were similar to isolates from South Africa (95.3%; accession no. MG255670) and Israel (96.4%; accession no. MN710208), respectively. Serotype 15 shared high similarity (98.3%) with an isolate from Israel (accession no. KP821098). On segment 2 gene phylogeny, BT viruses clustered into 12 nucleotypes. BT viruses detected in this study clustered into five distinct nucleotypes (i.e., B, E, F, G and J) (Figure 2). In Northern Province, we found nucleotypes E and F while in Eastern Province, nucleotypes F, B, and J were identified. In Western and Southern Provinces, nucleotypes E, G and G, F were detected, respectively.

**Figure 3:**
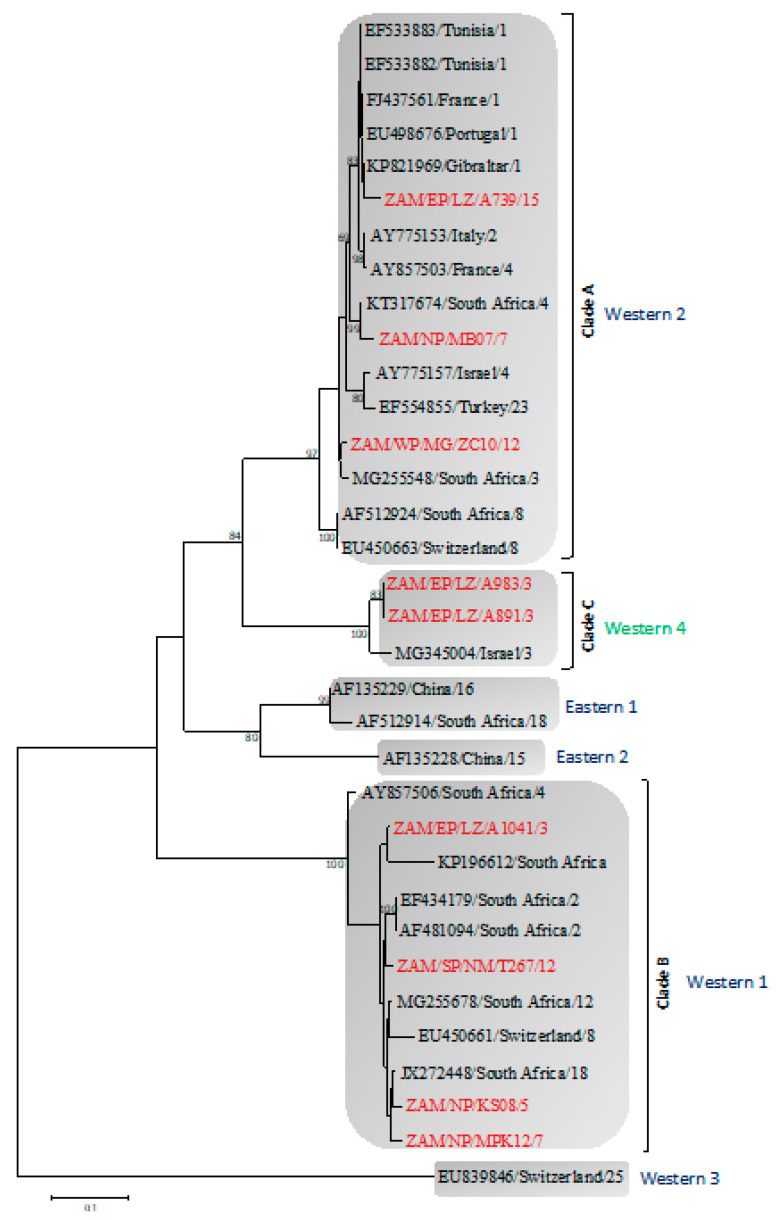
Phylogenetic tree of segment 10 gene of viruses detected in this study. The tree was generated using the Maximum Likelihood method based on the Tamura 3-parameter (T92) model with 1000 bootstrap replicates. Numbers at branch nodes indicate bootstrap values (> 60%). Viruses characterized in the present study are in red text. Novel topotype is in green text. Right brackets—clade. Shaded area—topotype. Reference sequences for Western and Eastern topotypes are in black text. Bar—number of nucleotide substitutions per site. Abbreviations: EP, Eastern Province; SP, Southern Province; WP, Western Province; NP, Northern Province; LZ, Lundazi; MG, Mongu; NM, Namwala; MB, Mbala; KS, Kasama; MPK, Mpika.

On NS3 gene phylogeny, BTVs clustered into Western and Eastern topotypes (Figure 3). Topologically, viruses from this study formed three distinct clades within the Western topotypes. Clade A consisted of serotype 7, 12 and 15 from the Northern, Eastern and Western provinces and clustered with Western 2 topotypes from South Africa and Gibraltar. Clade B included serotypes 3, 5, 7 and 12 from the Northern, Southern and Eastern provinces that were closely related to Western 1 topotypes from South Africa. Interestingly, clade C contained serotypes 3 and 7 from the Eastern Province, which along with an isolate from Israel, showed a phylogenetic clustering pattern closely related to, but distinct from the Western 2 topotype.

### 3.2. BTV Antibodies in Cattle

Antibodies against BTV were detected in 432 of 449 of the cattle tested by ELISA. Overall seroprevalence was high (96.2%, 95% CI (94.0–97.6)) for all study sites. In Chongwe District in Lusaka Province, 95.5% (105/110) of the cattle tested were seropositive for antibodies against BTV (95% CI, (89.8–98.0)). In Central Province, antibodies against BTV were detected in all the three study sites; Itezhi tezhi (92.1%; 95% CI (85.7–95.8)), Shibuyunji (98.2%; 95% CI (93.6–99.5)) and Mumbwa districts (99.1%; 95% CI (95.2–99.9)).

## 4. Discussion

Although mosquitoes are not competent vectors for BTV, the incidental detection of BTV nucleic acid from a blood-engorged pool collected from Western Province of Zambia [31] supports the idea of a potential role of mosquitoes in the epidemiology of BT [32,33]. Detection of BTV nucleic acid and antibodies in indigenous cattle and goats in eight provinces (i.e., Eastern, Copperbelt, Southern, Northern, Muchinga, Western, and Lusaka and Central) indicates widespread distribution of BT in Zambia. There was a significant difference (*p* < 0.0001, OR = 5.4) in nucleic acid prevalence rates between the wet and dry season (7.3% vs. 2.2%; Table 3), which is likely attributable to increased vector activity in the wet season [33]. In addition, this finding suggests seasonal cyclicity of BT in Zambia. The prevalence of BTV nucleic acid observed in cattle and goats in both wet (7.3%) and dry (2.7%) seasons as compared to wildlife (0.4%), suggests that cattle and goats, unlike wildlife, play an important role in the maintenance of BTV in Zambia. These results support earlier assertions that cattle and small ruminants such as goats, are important reservoir hosts for BTV in Africa [33,34,35].

Even though our results suggest a limited role of wild ruminants in the epidemiology of BT in Zambia, at the wildlife–livestock interface area, the role of wildlife in disease maintenance and transmission must not be overlooked. A previous study in Zambian wild ruminants demonstrated presence of antibodies against BTV [20]. Similarly, high prevalence of antibodies against BTV were demonstrated in wildlife in Zimbabwe (44.1%; 353/800) and Botswana (71.5%; 348/487) [36,37], intimating a wildlife role in the maintenance of BTV. To further clarify the role of wildlife in the epidemiology of BT in Zambia and the region, more studies will need to be conducted at the wildlife–livestock interface areas.

In this study, BTV nucleic acid in a local breed of sheep from Mongu District in Western Province was not detected, although goats from the same herd were positive for BTV nucleic acid. This was possibly due to virus clearance and/or lack of exposure to BTV prior to sampling. Notably, the farmer did not experience a disease in sheep with clinical signs similar to BT, probably due to the fact that indigenous local breeds, unlike exotic ones, are resistant to BT [38]. Still, this finding was intriguing as sheep are known to be generally more susceptible to BTV infection than goats [3].

Five different BTV serotypes were detected in cattle, that is 3, 5, 7, 12 and 15, while only serotype 5 was detected in goats. This heterogeneity of serotypes suggests that cattle, unlike goats, could be the main reservoir hosts for BTV in Zambia. Serotypes 3 and 15 detected in Eastern Province were previously reported in neighboring Malawi [19]. Similarly, in the segment 10 phylogeny (Figure 3), most viruses detected in this study were closely related to isolates from South Africa. This finding suggests possible transboundary exchange of viruses through legal and/or illegal trade of livestock. Accordingly, the detection of BTV nucleic acid and multiple circulating serotypes, particularly in regions bordering other countries (Figure 1), stresses the need for a regional effort in the control and prevention of BT. The identification of five different serotypes and three Western topotypes intimates a high genetic diversity among BTV in Zambia. Two viruses (i.e., ZAM/EP/LZ/A983/3 and ZAM/EP/LZ/A891/3) in clade C (Figure 3) clustered distinctly from the closely related Western 2 topotype. From a previously published report [14], close inspection of two nucleotide sequences from Eastern 1 topotype (accession no. AF135229) and Eastern 2 topotype (accession no. AF135228) revealed 87.3% nucleotide similarity. A reference sequence from Western 2 topotype (accession no. EF554855) and the two viruses from this study in clade C showed 85.9–86.1% nucleotide similarity. Based on this analogy, we propose this group to be a novel topotype and have thus designated it as Western 4 topotype.

The seroprevalence (96.2%) of BTV in cattle from Lusaka and Central provinces was high, indicative of widespread exposure to infection. High seroprevalence (95.9%) has been previously reported in cattle from Madagascar [35]. The observed high seroprevalence in cattle from Zambia was possibly due to increased *Culicoides* activity since sampling was undertaken during the wet season. This finding supports our results of high nucleic acid prevalence in the wet season (Table 2). Taken together, molecular and serologic results suggest possible BT endemicity in Zambia. The relatively low sheep population (*n* = 170,262) has likely contributed to the silent circulation of the virus. Nevertheless, the increased preference for BT-susceptible exotic breeds of sheep, goats and cattle during restocking may lead to the emergence of BT [3]. Recently, there has been a high demand for small ruminants in the region with other markets emerging in the Middle East. Most small- and large-scale farmers in Zambia have exploited the available regional markets for small ruminants. Furthermore, the transfrontier increase in movement of livestock that is driven by price differences, socio-cultural links, climate change, transhumance and nomadism [39], necessitates the need for regional BT prevention and control strategies that will mitigate potential socio-economic effects.

## 5. Conclusions

This study has demonstrated the presence of BTV nucleic acid and antibodies in domestic and wild ruminants in Zambia. Findings from this study have national and regional implications for the control and prevention of BT. It is anticipated that this work will not only improve the understanding of BT, but will also contribute to the formulation of evidence-based prevention and control strategy/policy in Zambia and the wider region.

## Figures and Tables

**Table 1:** Summary of blood and serum samples collected from domestic and wild ruminants in selected provinces Zambia.

Province	District	Species	Blood ^†^	Sera	Year (Season)
Copperbelt	Mpongwe	Cattle	100 (10)	-	2018 (Dry)
Eastern	Lundazi	Cattle	350 (35)	-	2018 (Wet)
Northern	Mbala	Cattle	200 (20)	-	2016 (Wet)
	Kasama	Cattle	243 (25)	-	2016 (Wet)
	Mpika	Cattle	200 (20)	-	2016 (Wet)
North-Western ^‡^	Ikelenge	Buffalo	5(1)	-	2017 (Dry)
Western	Mongu	Cattle	210 (21)	-	2018 (Dry)
		Goats	84 (9)	-	2018 (Dry)
		Sheep	6 (1)	-	2018 (Dry)
	Senanga	Cattle	350 (35)	-	2017 (Dry)
Southern	Livingstone	Cattle	104 (11)	-	2019 (Dry)
	Livingstone	Goats	99 (10)	-	2019 (Dry)
	Livingstone	Buffalo	11 (5)	-	2017 (Dry)
	Namwala	Cattle	188 (19)	-	2017 (Wet)
Kafue National Park	Mumbwa	Impala	98 (21)	-	2017 (Dry)
		Hartebeest	29 (8)	-	2017 (Dry)
Lusaka ^‡^	Chongwe	Hartebeest	76 (8)	-	2018 (Dry)
Central	Shibuyunji	Cattle		110	2017 (Dry)
	Mumbwa	Cattle		115	2017 (Wet)
	Itezhi tezhi	Cattle		114	2017 (Wet)
Lusaka	Chongwe	Cattle		110	2017 (Wet)
Total			2353 (259)	449	

^†^ Total number of samples (pooled samples), ^‡^ private game ranch.

**Table 2:** Bluetongue virus nucleic acid detection results in pooled blood from domestic and wild ruminants in selected provinces of Zambia.

Province	District	Species	Pools Tested	qRT-PCR ^†^	Season
Copperbelt	Mpongwe	Cattle	10	1 (10)	Dry
Eastern	Lundazi	Cattle	35	16 (45.7)	Wet
Northern	Mbala	Cattle	20	16 (80)	Wet
	Kasama	Cattle	25	18 (72)	Wet
	Mpika	Cattle	20	11 (55)	Wet
North-Western ^‡^	Ikelenge	Buffalo	1	0 (0)	Dry
Western	Mongu	Cattle	21	4 (19)	Dry
		Goats	9	2 (22.2)	Dry
		Sheep	1	0 (0)	Dry
	Senanga	Cattle	35	2 (5.7)	Dry
Southern	Livingstone	Cattle	11	6 (54.5)	Dry
		Goats	10	8 (80)	Dry
		Buffalo	5	0 (0)	Dry
	Namwala	Cattle	19	2 (10.5)	Wet
Kafue National Park	Mumbwa	Impala	21	0 (0)	Dry
		Hartebeest	8	1 (12.5)	Dry
Lusaka ^‡^	Chongwe	Hartebeest	8	0 (0)	Wet
Total			259	87 (33.6)	

^†^ Positive (percent), ^‡^ private game ranch.

**Table 3:** Pooled prevalence of bluetongue virus nucleic acid during different seasons in domestic and wild ruminants from Zambia.

Species	Sampling Season	Pools ^†^	Pooled Prevalence ^‡^
Cattle, goats, buffalo, impala, hartebeest	Wet, Dry	87/259 (33.6)	4.4 (3.6–5.4)
Cattle, goats, buffalo, impala, hartebeest	Dry	17/99 (17.2)	2.2 (1.4–3.2)
Cattle	Wet and Dry	76/196 (38.8)	4.8 (3.8–6.0)
Cattle	Wet	63/119 (52.9)	7.3 (5.6–9.3)
Cattle	Dry	13/77 (16.9)	1.8 (1.0–3.1)
Goats	Wet	-	-
Goats	Dry	10/19 (52.6)	7.2 (3.3–13.1)
Cattle and Goats	Dry	23/96 (24.0)	2.7 (1.7–4.0)
Wildlife	Dry	1/43 (2.3)	0.4% (0.0–1.8])

^†^ Positive pools/total no. tested (% positive), ^‡^ percent (95% CI); %, percent; CI, confidence interval.
